# 6-Gingerol Inhibits De Novo Lipogenesis by Targeting Stearoyl-CoA Desaturase to Alleviate Fructose-Induced Hepatic Steatosis

**DOI:** 10.3390/ijms252011289

**Published:** 2024-10-20

**Authors:** Pan Li, Tingting Wang, Hongmei Qiu, Ruoyu Zhang, Chao Yu, Jianwei Wang

**Affiliations:** 1 Chongqing Key Laboratory for Pharmaceutical Metabolism Research, Chongqing Medical University, Chongqing 400016, China; lipan2019@cqmu.edu.cn (P.L.); wangtingting@cqmu.edu.cn (T.W.); 2 College of Pharmacy, Chongqing Medical University, Chongqing 400016, China; qiuhongmei@cqmu.edu.cn; 3 Chongqing Key Laboratory of Traditional Chinese Medicine for Prevention and Cure of Metabolic Diseases, Chongqing Medical University, Chongqing 400016, China; ruoyuzhang@hkbu.edu.hk; 4 School of Chinese Medicine, Hong Kong Baptist University, Kowloon Tong, Hong Kong 999077, China

**Keywords:** 6-gingerol, metabolic-associated fatty liver disease, stearoyl-CoA desaturase, target, hepatic steatosis

## Abstract

Metabolic-associated fatty liver disease (MAFLD), also known as non-alcoholic fatty liver disease (NAFLD), is a worldwide liver disease without definitive or widely used therapeutic drugs in clinical practice. In this study, we confirm that 6-gingerol (6-G), an active ingredient of ginger (*Zingiber officinale Roscoe*) in traditional Chinese medicine (TCM), can alleviate fructose-induced hepatic steatosis. It was found that 6-G significantly decreased hyperlipidemia caused by high-fructose diets (HFD) in rats, and reversed the increase in hepatic de novo lipogenesis (DNL) and triglyceride (TG) levels induced by HFD, both in vivo and in vitro. Mechanistically, chemical proteomics and cellular thermal shift assay (CETSA)–proteomics approaches revealed that stearoyl-CoA desaturase (SCD) is a direct binding target of 6-G, which was confirmed by further CETSA assay and molecular docking. Meanwhile, it was found that 6-G could not alter SCD expression (in either mRNA or protein levels), but inhibited SCD activity (decreasing the desaturation levels of fatty acids) in HFD-fed rats. Furthermore, SCD deficiency mimicked the ability of 6-G to reduce lipid accumulation in HF-induced HepG2 cells, and impaired the improvement in hepatic steatosis brought about by 6-G treatment in HFD supplemented with oleic acid diet-induced SCD1 knockout mice. Taken together, our present study demonstrated that 6-G inhibits DNL by targeting SCD to alleviate fructose diet-induced hepatic steatosis.

## 1. Introduction

Metabolic-associated fatty liver disease (MAFLD), formerly known as non-alcoholic fatty liver disease (NAFLD), affects a quarter of the adult population and has no definitive or widely used therapeutic drugs for clinical use worldwide [1,2]. MAFLD can progress from initial simple hepatic steatosis to non-alcoholic steatohepatitis (NASH), fibrosis, cirrhosis, and eventually, hepatocellular carcinoma [3]. The pathogenesis of MAFLD is complicated, and it carries a significant risk for the development of diabetes, obesity, cardiovascular diseases and tumors [1,4]. Thus, there is an urgent need for basic theoretical research and drug development for MAFLD.

Stearoyl-CoA desaturase 1 (SCD1), also known as SCD in homo sapiens and rattus norvegicus, located in the endoplasmic reticulum (ER), is a key enzyme regulating the synthesis and metabolism of fatty acids. SCD is involved in hepatic lipogenesis and the beta-oxidation of fatty acids by catalyzing saturated fatty acids to unsaturated fatty acids and inhibiting fatty acids from entering the mitochondrion [5,6]. Clinical evidence suggested a positive correlation between the incidence of MAFLD and SCD desaturation indices [7,8]. Moreover, both the high expression and activity of SCD were observed in the liver of hamsters fed high-fat, high-fructose and high-cholesterol diets [9]. In nutrient-induced SCD1 knockout (*SCD1^−/−^*) mice, it was indicated that the AMP-activated protein kinase (AMPK) signaling pathway was activated, leading to a reduction in acetyl-CoA carboxylase (ACC) activity and an increase in carnitine palmitoyltransferase-1 (CPT1), which alleviates hepatic lipid accumulation [10,11]. Existing evidence indicates that high expression or strong activity of SCD induces the development of MAFLD, diabetes and obesity. Therefore, targeting SCD may be an effective strategy and a promising approach for the treatment of metabolic diseases such as MAFLD.

Traditional Chinese medicine (TCM), as a conventional and effective therapeutic strategy, has been widely used to treat metabolic diseases such as MAFLD in clinical settings for thousands of years [12,13]. Ginger (*Zingiber officinale Roscoe*) is one of the most commonly used TCMs or spices in China, and modern pharmacological studies have shown that the raw extract of ginger exhibits various pharmacological activities such as anti-oxidant, anti-cancer and lipid-lowering effects [14,15,16]. In particular, 6-gingerol (6-G), a major polyphenol component of ginger, was shown to reduce nutrient-induced hepatic steatosis in rodents [17,18,19]. However, the underlying mechanism of 6-G is not yet fully understood, particularly its direct binding target(s) in the improvement of MAFLD, which requires further in-depth exploration.

Dietary management is pivotal in mitigating the progression of metabolic diseases. Calorie restriction, along with high-protein or low-carbohydrate diets, are widely acknowledged as effective dietary interventions for patients with MAFLD [20]. Fructose, a simple sugar abundant in fruits and vegetables, is commonly used as a sweetener in many processed foods. Despite its reputation as a “healthy” sugar, excessive consumption of foods rich in fructose (such as high fructose corn syrup, soft drinks, juice, and baked goods) has been linked to negative health outcomes, including increased triglyceride levels, the development of MAFLD, obesity, insulin resistance and diabetes, especially in children and adolescents [21,22,23]. Overall, high-fructose intake stands as a significant risk factor for the onset and progression of metabolic diseases (such as MAFLD) in modern society.

In this study, we aimed to investigate the role of 6-G in fructose-induced hepatic steatosis, identify its direct binding targets, and to explore further the related molecular mechanisms. Our findings confirmed that 6-G can reduce hepatic steatosis both in vivo and in vitro. Using the chemical proteomics and cellular thermal shift assay (CETSA)–proteomics approaches, we identified SCD as one of the key direct-binding targets of 6-G. This finding was further verified by CETSA assay and molecular docking analysis. We found that 6-G suppressed SCD activity in high fructose diet (HFD)-fed rats, without altering SCD expression, which may be attributed to the direct binding effect of 6-G and SCD. Furthermore, the knockdown of SCD mimicked the improving lipid accumulation activity of 6-G in high fructose (HF)-induced HepG2 cells. And we also found that 6-G could not alleviate hepatic lipid accumulation in HFD plus oleic acid (OA) supplemented diet-induced *SCD1^−/−^* mice. This evidence suggests that 6-G alleviates HF-induced hepatic steatosis by targeting SCD. Overall, our present study provides a new mechanistic insight into the bio-activity of 6-G in alleviating hepatic steatosis.

## 2. Results

### 2.1. 6-G Lows the Lipid Accumulation in HF-Induced HepG2 Cells

**Figure 1:**
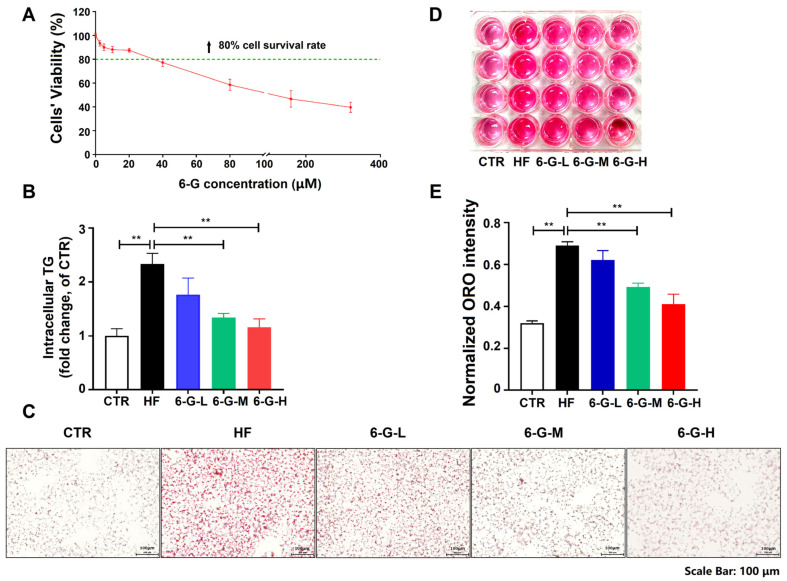
6-G reduces the lipid accumulation in HF-induced HepG2 cells. (**A**) MTT assay. (**B**) Cellular TG. (**C**) ORO staining of HepG2 cells. (**D**) ORO staining of cellular extract of HepG2 cells in DMSO. (**E**) OD intensity of the cellular extract with ORO staining at wavelength of 490 nm. 6-G-L: 5 μM 6-G; 6-G-M: 10 μM 6-G; 6-G-H: 20 μM 6-G. Data are expressed as mean ± SD, *n* = 3 independent experiments. ** *p* < 0.01.

A dose of 20 mM fructose (HF) was used to induce HepG2 cells to evaluate the effects of 6-G on fructose-induced hepatic lipid accumulation in vitro. The MTT assay indicated that 6-G showed no obvious cytotoxicity, with cell survival rates reaching 80% at concentrations ranging from 0 to 20 μM (Figure 1A). Furthermore, 6-G (from 5 to 20 μM) decreased cellular triglyceride (TG) levels in a dose-dependent manner, with significant reductions observed at 10 and 20 μM in HF-induced HepG2 cells (Figure 1B). Oil Red O (ORO) staining also confirmed that 6-G could obviously suppress the increase in lipid droplets caused by HF (Figure 1C–E). These findings suggest that 6-G reduces lipid accumulation in HF-induced HepG2 cells.

### 2.2. 6-G Ameliorates Metabolic Syndrome and Hepatic Steatosis in HFD-Induced MAFLD Rats

**Figure 2:**
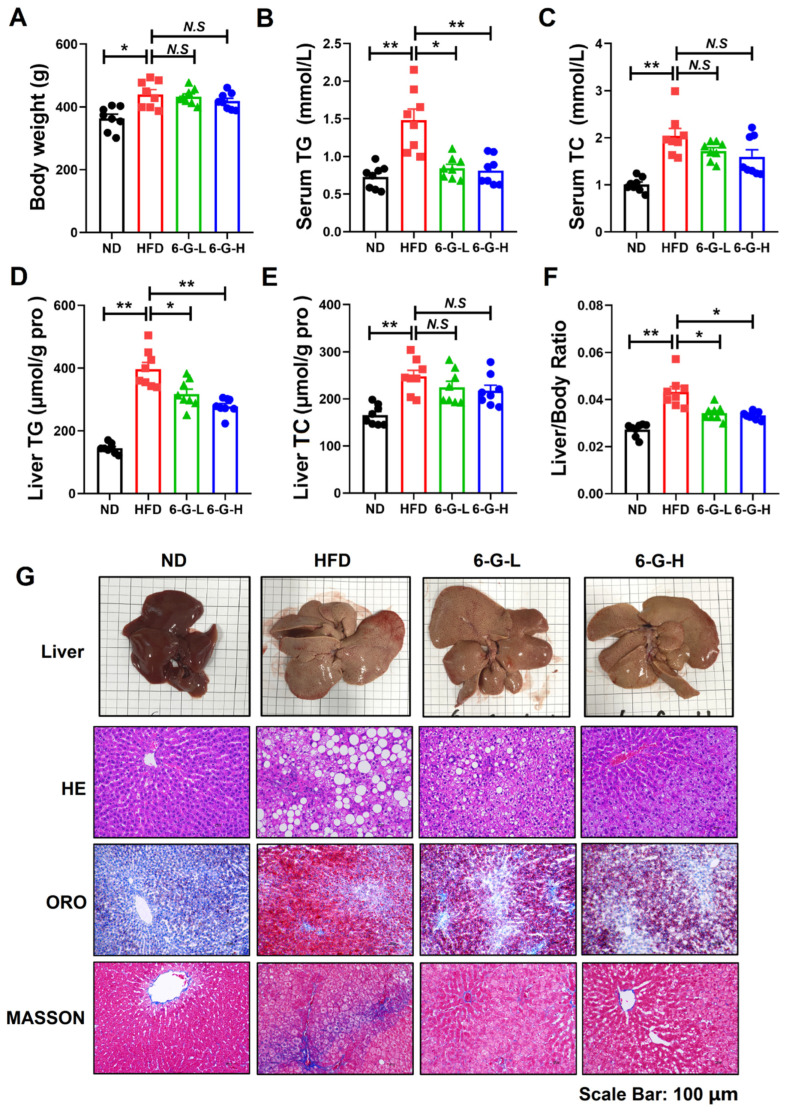
6-G ameliorates metabolic syndrome and hepatic steatosis in HFD-induced disease in rats. (**A**) Body weight. (**B**) Serum TG. (**C**) Serum TC. (**D**) Liver TG. (**E**) Liver TC. (**F**) Ratio of liver/body weight in rats. (**G**) Liver pathology of rats, including liver images and HE, ORO, Masson staining. ND: normal diet; HFD: high-fructose diet; 6-G-L: 0.1 mg/kg; 6-G-H: 0.4 mg/kg. Data are expressed as mean ± SEM, *n* = 8 rats per group. ** *p* < 0.01, * *p* < 0.05. N.S: no significant differences.

HFD-induced MAFLD rats were established to evaluate the effect of 6-G on metabolic syndrome and hepatic steatosis in vivo. The results showed that 6-G treatment did not alter the body weight (Figure 2A) or chow intake (Figure S1A) of rats compared with pure HFD-fed rats. Pure HFD-fed rats exhibited obviously increased levels of blood fats, including serum TG, TC and LDL-c, along with decreased HDL-c. However, 6-G treatment reduced serum TG (Figure 2B), but had no significant effect on serum TC (Figure 2C), LDL-c and HDL-c (Figure S1B,C). As expected, 6-G significantly alleviated hepatic steatosis in HFD-fed rats, evidenced by reducing liver TG (Figure 2D), decreasing liver/body weight ratio (Figure 2F), causing fewer and smaller lipid droplets and lower collagen deposition in the liver (Figure 2G). Additionally, 6-G treatment had little effect on liver TC (Figure 2E). Overall, these findings manifest the beneficial effects of 6-G on improving metabolic syndrome and hepatic steatosis in HFD-fed rats.

### 2.3. 6-G Directly Binds to SCD in MAFLD Cells

**Figure 3:**
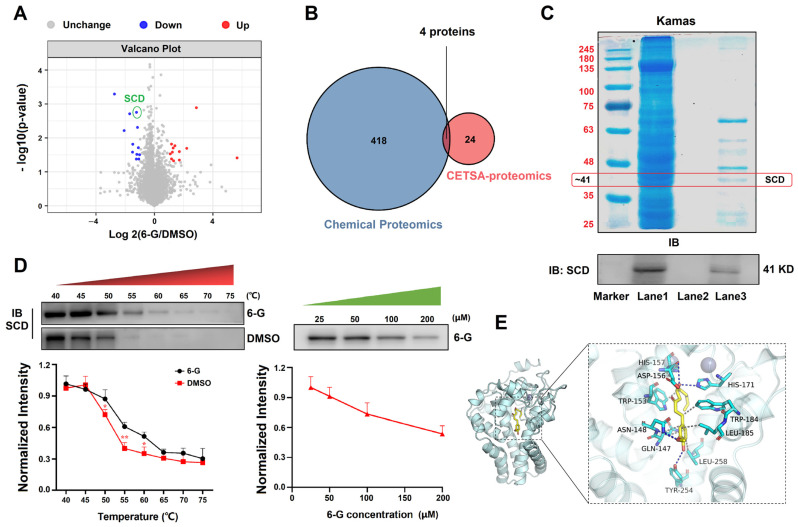
6-G binds directly to SCD. (**A**) Differentially expressed proteins identified by CESTA-integrated quantitative proteomics analysis between 6-G and DMSO treatments. (**B**) Venn analysis of the targets identified by chemical proteomics and CETSA proteomics. (**C**) Efficiency evaluation of magnetic capture (chemical proteomics) by SDS-PAGE. The upper image displays Coomassie blue staining of SDS-PAGE while the lower image shows Western blot verification. Lane 1 represents the lysate of HF-induced HepG2 cells as a loading control, lane 2 exhibits the lysate captured by the functionalization of azide-MMs as a negative control, lane 3 indicates the lysate captured by the Al-6G-MMs. (**D**) CETSA assay for validation. The left panel shows SCD protein levels after heat treatment at different temperatures, the right panel shows SCD levels after treatment with different concentrations of 6-G at 55 °C. (**E**) Molecular docking. The left image exhibits the overall view for docking and the right image shows the detailed view. The yellow stick represents 6-G molecule, blue carton represents SCD protein, blue lines represent hydrogen bonding, and gray dashed lines stand for hydrophobic interactions. Data are expressed as mean ± STD, *n* = 3 independent experiments. ** *p* < 0.01, * *p* < 0.05.

Given that 6-G alleviates hepatic steatosis both in vitro and in vivo, the direct binding targets of 6-G were identified by two methods to elucidate the deeply molecular mechanism underlying its effects on improving MAFLD. By chemical proteomics for target fishing (data for characterizing of AI-6G by ^1^H-NMR and ^13^C-NMR are shown in Supplemental Figure S2), it was indicated that 6-G directly bound to 422 proteins in HF-induced HepG2 cells, including PHKB, SCD, RFK, SOAT2, PRKACG, etc., (a complete list has not been shown due to our future study on the direct targets and other biological activities of 6-G). Further functional annotations using KEGG pathway enrichment analysis revealed that these 422 protein targets were mainly enriched in the regulation of metabolic pathways, glycolysis/gluconeogenesis, fatty acid degradation, fatty acid metabolism and glucagon signaling pathway (Figure S3A). To improve the accuracy of target identification, CESTA-integrated quantitative proteomics was also employed for target identification. This analysis identified a total of 24 differentially expressed proteins in the comparative treatment of 6-G and DMSO (fold change > 2 and *p* < 0.05), including SCD, SOAT2, DBI, PHKB, PRKACG, etc., (the 24 differentially expressed proteins are shown in Supplemental Table S1); among these proteins, 12 proteins were upregulated and 12 were downregulated by 6-G treatment, indicating potential direct binding targets of 6-G (analyses of volcano map, statistical bar chart and GO enrichment are shown in Figure 3A, Figure S3B,C, respectively). More importantly, further analysis intersected the targets obtained from both identification methods, revealing a total of four targets, including SCD, were the final identified targets of 6-G (Figure 3B).

As described previously, SCD is a key enzyme regulating the synthesis and metabolism of fatty acids, making it a potential target of 6-G in regulating hepatic fatty acid metabolism. Therefore, we performed experimental verification for this target. To reconfirm the direct binding effect of 6-G and SCD, Western blot, CETSA and molecular docking were designed in the subsequent research. The results of Coomassie blue staining (CBS) showed the differences between these three lanes in the gels, indicating that some proteins were fished by Al-6G. It was found that the molecular weight appeared at 25 KD, 29 KD, 41 KD, 46 KD, 55 KD, 59 KD and 190 KD, in the “Al-6G-fished” lane. Subsequent Western blot analysis confirmed that the fished protein (approximately 41 KD) was SCD (Figure 3C). Meanwhile, CETSA analysis indicated that 6-G significantly changed the thermal stability of SCD in a dose-dependent manner (Figure 3D). Molecular docking showed the binding energy between 6-G and SCD was −7.559 kcal/mol, revealing a hydrogen bonding (ASN-148, TYR-254, HIS-171, ASP-156) and hydrophobic interaction (LEU-258, HIS-157, LEU-185, TRP-184, GLN-147, TRP-153) between SCD and 6-G (Figure 3E). Taken together, these results suggest that 6-G directly binds to SCD, confirming that SCD is a direct binding target of 6-G.

### 2.4. 6-G Inhibits SCD Activity Without Affecting SCD Expression

**Figure 4:**
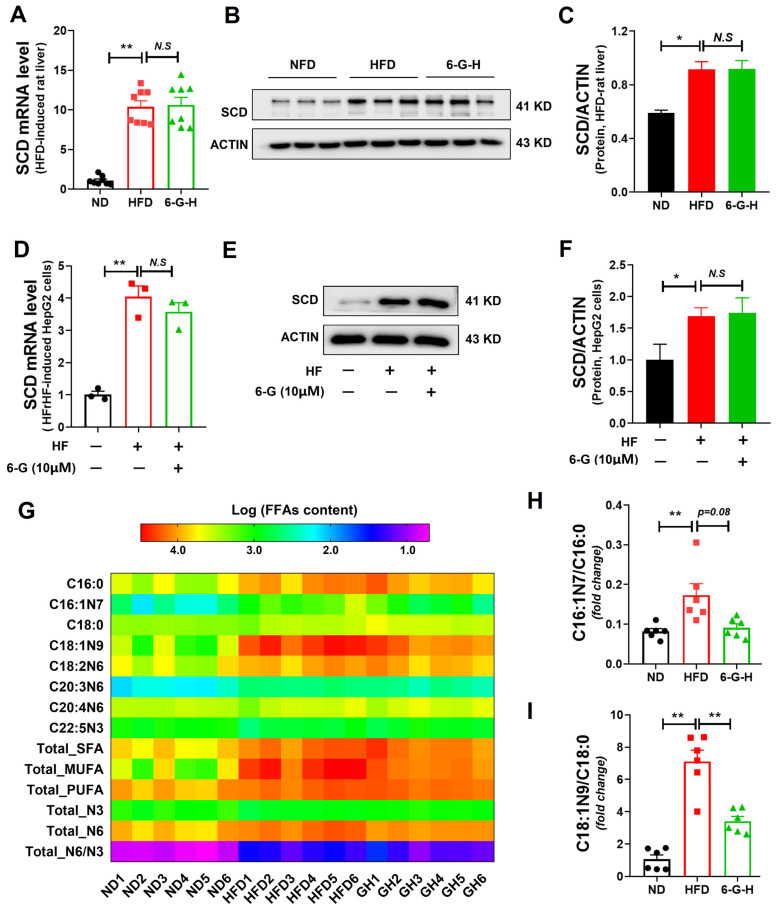
6-G suppresses SCD activity independently of SCD expression regulation. (**A**) Relative mRNA expression of SCD in rat livers. (**B**,**C**) SCD protein levels in rat livers. (**D**) SCD mRNA levels in HepG2 cells. (**E**,**F**) SCD protein expression in HepG2 cells. (**G**) Heatmap of the representative FFAs. FFAs: free fatty acids. (**H**,**I**) Ratios of C16:1N7/C16:0, C18:1N9/C18:0 in rat livers, showing SCD activity. Data are presented as mean ± SEM (in vivo, *n* ≥ 6/group) and mean ± STD (in vitro, *n* = 3 independent experiments). ** *p* < 0.01, * *p* < 0.05, N.S: no significant differences.

Our findings indicate that 6-G directly binds to SCD as demonstrated above. We then explored the effects of this interaction on SCD activity and expression. Since SCD expression influences its activity and the associated biological processes, we first investigated the impact of 6-G on SCD expression in MAFLD. It was found that HFD feeding increased SCD expression in rat liver at both the mRNA and protein levels. Interestingly, the 6-G treatment did not significantly alter SCD expression (Figure 4A–C). Similar results were also observed in HF-induced HepG2 cells (Figure 4D–F).

Furthermore, medium-chain and long-chain fatty acids in the livers of HFD-fed rats were measured by targeted metabolomics. The representative content of free fatty acids is shown in Figure 4G and Supplemental Table S2. The results indicated that HFD-feeding increased the hepatic levels of C16:0 (methyl palmitate), C16:1N7 (methyl palmitoleate), C18:0 (methyl stearate), C18:1N9 (methyl oleate), etc., while 6-G treatment reduced the C16:1N7 and C18:1N9 levels. Significantly, further analysis revealed that HFD-feeding induced an increase in the C16:1N7/C16:0 and the C18:1N9/C18:0 ratios were abolished by 6-G treatment, suggesting that 6-G suppresses hepatic SCD activity in HFD-fed rats (Figure 4H,I). Meanwhile, 6-G did not affect the C18:0/C16:0 and C18:1N9/C16:1N7 ratios, implying that the elongation activity of fatty acids (mainly regulated by the ELOVL fatty acid elongase family) was not modified by 6-G treatment in rat liver (Figure S4A,B). Altogether, these findings manifest that 6-G inhibits SCD activity independently of SCD expression regulation. The inhibition is likely due to the direct binding of 6-G to SCD, thereby suppressing SCD activity.

### 2.5. 6-G Attenuates SCD-Mediated DNL and Lipid Accumulation in HF-Induced HepG2 Cells

**Figure 5:**
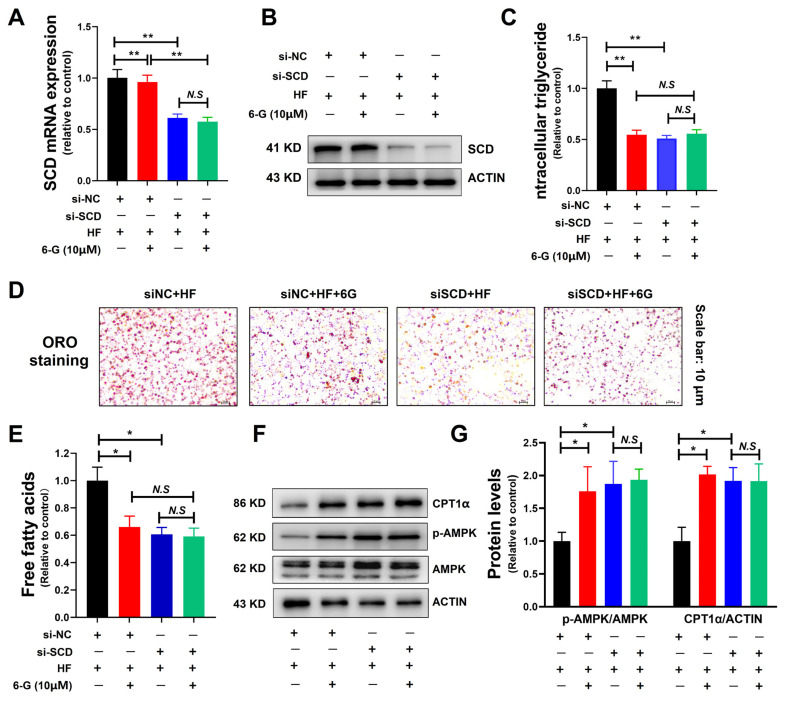
6-G alleviates SCD-mediated DNL and lipid accumulation in HF-induced HepG2 cells. (**A**,**B**) Knockdown efficiency of SCD at mRNA and protein levels by qRT-PCR and Western blot. (**C**) Relative expression of intracellular TG content to control (si-NC). (**D**) ORO staining. (**E**) Relative levels of intracellular free fatty acids to control. (**F**,**G**) Effect of SCD knockdown on AMPK and CPT1α levels, with or without 6-G treatment. siNC: blank siRNA, siSCD: knockdown of SCD by siRNA. Data are presented as mean ± STD, *n* = 3 independent experiments. ** *p* < 0.01, * *p* < 0.05, N.S: no significant differences.

To identify the role of SCD activity in the reduction in lipid accumulation by 6-G, we employed siRNA-mediated SCD knockdown in HF-induced HepG2 cells. The efficacy of SCD silencing in HepG2 cells was confirmed by qRT-PCR and Western blot (Figure 5A,B). Strikingly, the reduction in lipid accumulation by 6-G treatment was blocked after SCD knock-down in HepG2, as indicated by intracellular TG levels (Figure 5C) and ORO staining (Figure 5D). Meanwhile, evidenced by the results of the free fatty acids test in HepG2 cells, it was observed that 6-G treatment mimicked the effect of SCD knockdown on reducing de novo lipogenesis (DNL)-mediated fatty acid synthesis (Figure 5E). Given that SCD knockout can activate AMPK and ACC in mice, the key regulatory factors in DNL and fatty acid metabolism [10,11], further Western blot analysis indicated that knockdown of SCD significantly increased the expressions of p-AMPK/AMPK and CPT1α, which was similar to the effect of 6-G treatment alone. Moreover, after transecting SCD siRNA in HepG2 cells, the effects of 6-G treatment was inhibited (Figure 5F,G). Taken together, these data manifest that SCD is a requisite factor for 6-G to mitigate DNL and lipid accumulation in HF-induced HepG2 cells.

### 2.6. 6-G Relieves Fructose-Induced Hepatic Steatosis of Mice in a SCD1-Dependent Manner

**Figure 6:**
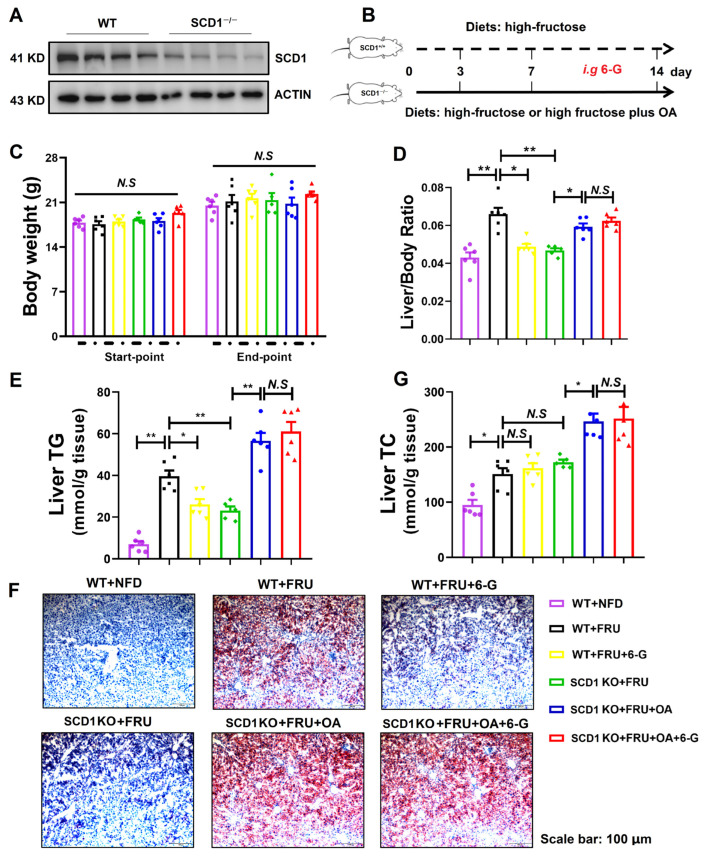
6-G improves fructose-induced hepatic steatosis of mice in a SCD1-dependent manner. (**A**) SCD1 expression in WT and *SCD1^−/−^* mice. (**B**) Scheme of mouse groupings and 6-G treatment. (**C**) Body weight of mice. (**D**) Ratio of liver/body weight. (**E**) Liver TG. (**F**) Liver ORO staining. (**G**) Liver TC. 6-G: 0.8 mg/kg. *n* ≥ 5 mice for each group. Data are expressed as mean ± SEM, ** *p* < 0.01, * *p* < 0.05, N.S: no significant differences.

The previous results indicate that 6-G attenuates SCD1-mediated DNL and lipid accumulation in HF-induced HepG2 cells. To further validate these findings, SCD1 knockout mice were employed. Hepatic steatosis was evaluated both in *SCD1^+/+^* mice (wild type, WT) and *SCD1^−/−^* mice. The SCD1 protein levels of WT and *SCD1^−/−^* mice were identified by Western blot (Figure 6A), and the experimental scheme is shown in Figure 6B. As expected, 6-G treatment did not affect the body weight among these mice groups (Figure 6C). Compared to the WT mice fed with fructose (WT + FRU), 6-G treatment (WT + FRU + 6-G) mimicked the reduction in liver/body ratio and liver TG seen in *SCD1^−/−^* mice with high fructose-feeding (*SCD1 KO* + FRU) (Figure 6D,E). Furthermore, it was found that the high-fructose diets supplemented with oleic acid (*SCD1 KO* + FRU + OA) could aggravate hepatic steatosis in *SCD1^−/−^* mice, evidenced by the increase in liver/body ratio and liver TG (Figure 6D,E). while the increased hepatic steatosis could not be relieved by 6-G, which was proved by further ORO staining (Figure 6F). Moreover, similar to the observations in rats fed by HFD, 6-G showed no significant effect on liver TC levels in either fructose-fed mice or fructose plus OA-fed mice (Figure 6G). Collectively, these findings suggest that 6-G alleviates fructose-induced hepatic steatosis in mice in a SCD1-dependent manner.

## 3. Discussion

In recent years, MAFLD has been a common condition with rising prevalence, and is becoming a growing global phenomenon [24,25]. It is well known that over-nutrition and excessive consumption of highly processed foods are the main initiating factors for the high prevalence of MAFLD. Currently, maintaining a healthy life style, including more exercise and dietary intervention, has been established as the main effective treatment for MAFLD. However, there are still some difficulties to be faced, as maintaining an exercise regimen is also a challenge. For the MAFLD patient, especially those with concurrent obesity, diabetes and even cardio-cerebral vascular diseases, adherence to regular exercise poses a particular challenge. On the other hand, changes to diet show a beneficial effect on relieving MAFLD, but it is still unclear what the most effective form of intervention is, as well as the related clear molecular mechanisms [26].

In the present study, we found that 6-G, the main active ingredient of ginger (*Zingiber officinale Roscoe*), used for medicinal purposes in TCM and as spice condiments to enhance flavor in cooking, could alleviate hepatic steatosis in HFD-induced MAFLD rats. Moreover, we found that 6-G also significantly attenuated hepatic DNL and lipid accumulation caused by high fructose both in vivo and in vitro. Specifically, in these animal models of MAFLD (caused by HF intake in rats and mice), it was observed that 6-G treatment caused reduced liver/body ratio, decreased liver lipid droplets and lowered liver TG content, while had no obvious effect on body weight or chow intake, manifesting that 6-G improved hepatic steatosis independent of weight loss and chow-intake reduction. Meanwhile, 6-G treatment showed no significant effects on TC either in the liver or the serum, which was consistent with our previous research in aging-induced hepatic steatosis rats [27,28]. These results indicate that 6-G exerts a stronger regulatory effect on TG compared to TC. Additionally, we chose a small dose of 6-G for the studies in rats and mice (0.1 to 0.8 mg/kg for 6-G, referenced by our previous studies as described above), further indicating the strong activity of 6-G in ameliorating MAFLD in vivo.

There are two main sources of fatty acids in the liver, including those derived from blood following lipolysis of TG in adipose tissue and those synthesized from glucose or fructose by DNL [29,30]. Evidence demonstrated that the increasing hepatic lipid content in NAFLD patients is largely attributed to DNL [31]. Meanwhile, dietary carbohydrates, especially fructose, are more potent inducers of DNL compared to glucose for converting excess carbons into lipids [32]. DNL is an endogenous lipid synthesis process from dietary sources (usually carbohydrates, or stored energy deposits), involving fatty acid synthesis, fatty acid elongation/desaturation, and triglyceride assembly. Among them, C16:0 (palmitate) is identified as the primary fatty acid synthesized endogenously, which can be elongated to the 18-carbon fatty acid stearate (such as C18:0, oleic acid) or to longer fatty acids by the enzyme elongation of the ELOVL fatty acid elongase family (mainly ELOVL6) [33,34]. And palmitoleate (16:1) and oleate (18:1) can be formed from palmitate and stearate by SCD catalysis, a key pathway for converting saturated to monounsaturated fatty acids during DNL. After desaturation, these fatty acids can be esterified to the glycerophosphate backbone to form the more complex lipids such as TG in the liver [32,33]. Elongation and desaturation (regulated by ELOVL6 and SCD activity, respectively) of long-chain fatty acids are the critical steps in hepatic DNL and fatty acid metabolism [35]. SCD is primarily regulated by high carbohydrate diets, and global SCD knockout significantly alleviates hepatic DNL, global SCD1 knockout significantly alleviates hepatic DNL and steatosis induced by high-carbohydrate high-fat diets in mice, and the beneficial protective effect can be blocked in liver-specific SCD1 knockout mice fed a high fructose with oleate acid supplementation diet [33,36,37,38]. Taken together, targeting SCD may be an effective strategy and a promising approach for ameliorating MAFLD.

In the current study, we identified that SCD may be a direct binding target of 6-G using both chemical proteomics and cellular thermal shift assay (CETSA)–proteomics. These two methods are well-recognized approaches for target identification as performed in previous studies [39,40,41], and of course, the different methods have their respective strengths and weaknesses. The chemical structure of the small molecule (ligand) is usually modified by chemical proteomics for target identification, which may cause the changes in bio-activity and original binding sites with the targets, leading to false positive identification [42]. Similarly, the CETSA–proteomics approach for identifying targets has some drawbacks, especially the control of conditions and accuracy of results, such as temperature control, criteria for screening of differential expressed proteins and the one-sidedness of evaluation results, etc., [43,44]. To improve the accuracy, we performed these two methods in our study for identifying the binding target of 6-G.

As we have shown, SCD is a direct binding target of 6-G; our subsequent experiments manifested that 6-G alleviates SCD-mediated DNL and lipid accumulation in HF-induced HepG2 cells, which was also verified in SCD^−/−^ mice fed high fructose with OA supplementation diets. These findings suggest that SCD plays an important role in relieving hepatic steatosis caused by high fructose supplementation, and it may be related to the inhibition of SCD-mediated DNL. Interestingly, it was found that 6-G did not affect the expression of SCD at both mRNA and protein levels, but suppressed SCD activity in HFD-fed rats, with further molecular docking also simulating this binding interaction. Suggesting 6-G inhibits SCD may be fully attributed to their direct binding interaction. Overall, 6-G inhibits DNL by targeting SCD to alleviate diet-induced hepatic steatosis.

## 4. Material and Methods

### 4.1. Cell Experiment

#### 4.1.1. Cell Culture, Modeling and Treatment

Human hepatocellular carcinoma cell line HepG2 (purchased from China Center for Type Culture Collection, Wuhan, China) was cultured in Dulbecco’s modified Eagle’s medium (DMEM, Gibco, Grand Island, NY, USA) containing 10% fetal bovine serum (Biological Industries, Kibbutz Beit Haemek, Israel) and supplemented with 10 mL/L penicillin-streptomycin (Gibco, New York, NY, USA). Samples of 1.2 × 10^4^ cells per well were planted in 96-well plates (for MTT assay) and 2 × 10^4^ cells per well were planted in 6-well plates (for lipid and protein detection). As previously reported with some modifications [45], for modeling the lipid accumulated in vitro, HepG2 cells were serum-starved for 24 h and treated with 20 mM fructose (Solarbio, Beijing, China). Then the cells were treated with various concentrations of 6-G (purity ≥ 98%, purchased from Shanghai Yuanye Biotechnology Co., Ltd., Shanghai, China. Dissolved in DMSO) for further bio-activity tests.

#### 4.1.2. MTT Assay

3-(4,5-Dimethylthiazol-2-yl)-2,5-diphenyltetrazolium bromide (MTT) was used to detect cell viability. After treatment with 6-G, the cells were incubated with 100 µL of regular medium with 50 µg MTT (Sigma-Aldrich, St. Louis, MO, USA) for 4 h and then dissolved in 150 µL DMSO (Shanghai Macklin Biochemical Co., Ltd., Shanghai, China) for measurement of absorbance by the microplate reader at 490 nm (Bio-Rad, Hercules, CA, USA).

#### 4.1.3. Triglyceride, Free Fatty Acid Detection, Oil Red O (ORO) Staining and Quantitation Assay in HepG2 Cells

To observe the lipid accumulation of HepG2 cells with 6-G treatment, we measured intracellular triglyceride (TG) content. After treatment, the cells were washed with PBS 3 times, then collected for TG detection using the Triglyceride Assay Kit (Applygen, Beijing, China), and free fatty acid measurement using Free Fatty Acids Assay Kit (Beyotime Biotechnology, Shanghai, China), strictly according to the instruction manual. TG content of the cells were normalized with its protein concentration.

Oil Red O (ORO) staining was also used to evaluate the lipid accumulation in HepG2 cells. Briefly, cells were washed with PBS buffer and fixed with 10% formalin for 30 min, then stained for 20 min with 100 µL of 0.5% ORO in isopropanol/water solution (3:2, *v*/*v*, Sigma-Aldrich). After that, the cells were differentiated in 75% ethanol for 2 min and washed with distilled water 5 times. Finally, the cells were imaged by an Image-Pro Plus (version 6.0, Media Cyberneyics, Inc., Rockville, MD, USA). To quantify the cellular lipids, 500 μL DMSO was added to dissolve the ORO dyes extracted from the stained cells, and the absorbance of DMSO extract was measured at 490 nm by the microplate reader.

#### 4.1.4. RNA Interference and Treatment

Cells were seeded into 6-well plates (2 × 10^5^ cells/well) and reached 75% confluence before transfecting with RNA Interference. The SCD siRNA (si-SCD) or control siRNAs (si-NC) were introduced into HepG2 cells using the lipofectamine 3000 transfection reagent according to the manufacturer’s instructions. RNA oligo was supplied by GenePharma (Beijing, China), and the sequences are shown in Supplemental Table S3. After 6 h, the transfection medium was removed and replaced with a complete medium, and the cells were cultured for 24 h, then the cells were treated with HF and 6-G as described in Section 4.1.1 for further study.

### 4.2. Animal Experiments

All animal experiments of the current study were accomplished in the Laboratory Animal Centre of Chongqing Medical University, China. All animal procedures were approved by the Institutional Animal Care and Use Committee of the Laboratory Animal Centre of Chongqing Medical University (approval NO: 2022093). All the experiments complied with the ARRIVE 2.0 guidelines and were performed in accordance with guidelines under the approval of the Animal Protection and Use Committee of Chongqing Medical University.

#### 4.2.1. High Fructose Diet-Induced Hepatic Steatosis Rats and 6-G Treatment

The 5–6-week-old male Sprague–Dawley (SD) rats (obtained from the Laboratory Animal Centre of Chongqing Medical University, Chongqing, China) were acclimatized with 12 h dark-light cycles under a constant temperature (24 ± 2 °C). After one week of adaptive feeding, rats were randomly divided into 4 groups, including normal-diet group (ND), high-fructose-diet group (HFD), 6-G-low-dose group (6-G-L) and 6-G-high-dose group (6-G-H). The ND group rats were fed with normal basic maintenance diet supplied by the Laboratory Animal Centre of Chongqing Medical University. HFD and 6-G treatment group rats were fed with HFD (20% food grade crystalline fructose was added to the ND diets, customized by Ensiweier Bioengineering Institute, Chongqing, China) for 12 weeks. Meanwhile, drug treatment started after 8-week dietary intervention and maintained for 4 weeks. Treatment scheme was as follows: 6-G-L and 6-G-H group rats were intragastrically administered with 0.1 mg/kg or 0.4 mg/kg 6-G (dissolved in 5% gum arabic solution) daily, respectively. ND and HFD group rats were supplied with the same volume solvent of gum arabic solution (5%). At the end of the experiment, rats were sacrificed under anesthesia by head dislocation and bio-samples were collected for further studies.

#### 4.2.2. Biochemical Assessment of Serum and Liver

Serum TG, total cholesterol(TC), low-density lipoprotein cholesterol (LDL-c), high-density lipoprotein cholesterol (HDL-c) in rats were measured using bio-chemical kits (purchased from Nanjing Jiancheng Bioengineering Institute, Nanjing, China). The liver TG and TC contents were detected by enzymatic assays kits (Applygen Technologies Inc., Beijing, China), and data were normalized by protein. All procedures were carried out according to the manufacturer’s instructions.

#### 4.2.3. Histological Assessment

Liver samples were fixed in 4% paraformaldehyde and embedded with paraffin, and samples were stained with hematoxylin eosin (HE), MASSON and ORO as our previous description [34]. Images of liver sections were captured with a light microscope (Nikon, Tokyo, Japan).

### 4.3. Target Identification

#### 4.3.1. Chemical Proteomics Approach

**Figure 7:**
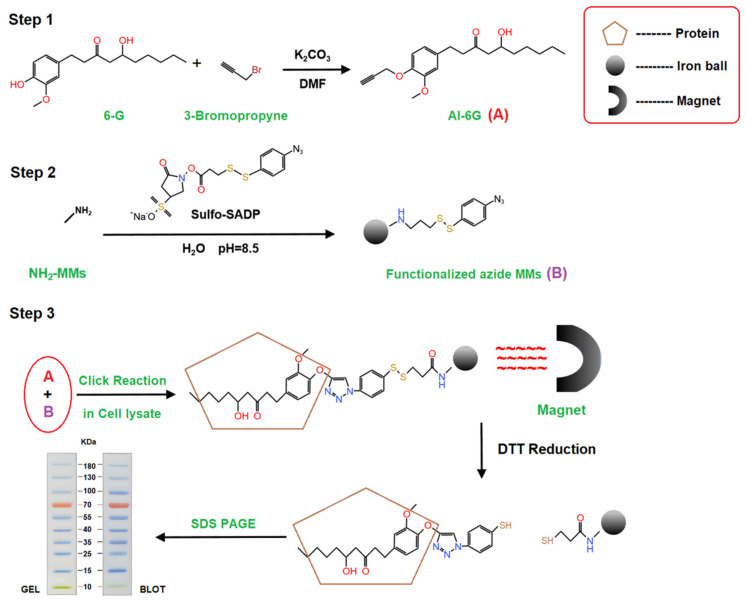
Experimental procedures of chemical proteomics approach to identify the direct binding targets of 6-G. DMF: N, N-Dimethylformamide; Sulfo-SADP: sulfosuccinimidyl (4-azido-phenyldithio) propionate.

A chemical proteomics approach was used for target identification in vitro as previously described [42]. Briefly, an alkynyl-6G probe (Al-6G) was synthesized and used to construct Al-6G functionalized magnetic microspheres (Al-6GMMs) for magnetic target fishing. HF-induced HepG2 cells were lysed with IP cell lysis buffer (Beyotime, Shanghai, China) to obtain the total protein lysis solution, and the lysis was incubated with 10 µM Al-6G probe for 6 h. After that, the suspension was treated with Al-6G-MMs and incubated with a catalyst system (100 µM sodium ascorbic acid and 100 µM CuSO4 in PBS) overnight at 4 °C, and then the Al-6G-MMs were separated with magnets. After washing, the enriched micro-spheres were treated with 100 µM dithiothreitol (DTT, Sigma-Aldrich, St. Louis, MO, USA) to obtain the target proteins of 6-G. SDS PAGE was performed to separate the proteins and Western blot was used to define the targets; the gels of SDS-PAGE were identified by HPLC-mass spectrometry (entrusted to Apply Protein Technology, Shanghai, China). The steps of target identification by the chemical proteomics approach are summarized in Figure 7.

#### 4.3.2. CETSA-Integrated Proteomics Analysis

**Figure 8:**
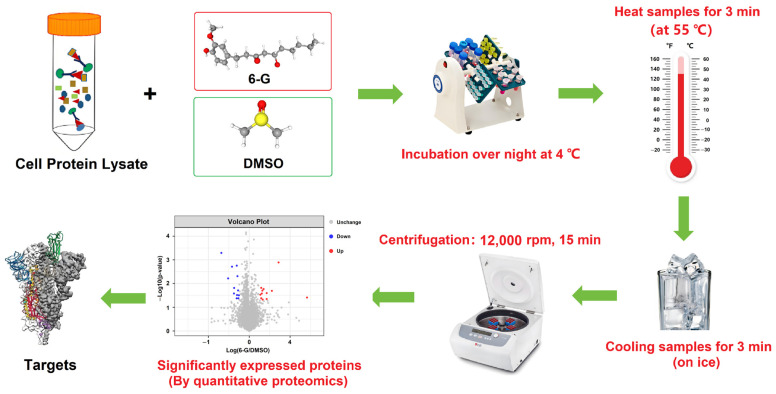
Technology roadmap of CETSA-integrated proteomics. Cell lysate was obtained from HF-induced HepG2 cells.

On the other hand, cellular thermal shift assay (CETSA)-integrated quantitative proteomics was also used for target identification [43,46]. As previously described, with some modifications, in reference [43], the cell lysate from HF-induced HepG2 cells was divided into two aliquots and treated with DMSO and 6-G overnight at 4 °C, respectively. Afterwards, these two aliquots were heated for 3 min at 55 °C (according to the pre-experimental results, the protein concentration for heat treatment at 55 °C showed a maximum statistical difference between the cell lysate treated with DMSO and 6-G) and then cooled on ice for 3 min immediately. The supernatants (after high-speed centrifugation) were analyzed for the differential proteins by quantitative proteomics analysis (entrusted to Apply Protein Technology, Shanghai, China). Western blot was used to identify the target after the supernatants were separated by SDS-PAGE. The experiments were repeated three times. The technology roadmap for CETSA-integrated proteomics is shown in Figure 8.

#### 4.3.3. CETSA Assay for Verification

CETSA assay was performed to validate the binding interaction between 6-G and SCD. Samples were prepared as described in Figure 8. After incubation over night at 4 °C, the lysate (including both DMSO and 6-G treatments) was divided into 8 groups and subjected to heat treatment at 40, 45, 50, 55, 60, 65, 70 and 75 °C, respectively. Then the supernatants obtained after centrifugation were used to assess SCD protein levels by Western blot. Additionally, a dose-dependent CETSA assay was conducted where the lysate was treated with varying concentrations of 6-G and heat-treated at 55 °C. The following Western blot analysis of SCD was performed as described above.

#### 4.3.4. Molecular Docking

Molecular docking was also used to test the binding activity and predict the possible binding sites of 6-G and SCD. The structure of SCD was obtained from PDB database (PDB ID: 4zyo), and 6-G was used to dock into SCD by AutoDock Vina 1.2.3. Then, the binding parameters and sites were obtained for analysis and results were visualized by PyMol 2.5.5.

### 4.4. SCD Activity Assay by Targeted Metabolomics

SCD activity in rat liver was also evaluated using targeted metabolomics as described in our previous study [34]. Briefly, 30 mg of liver tissues were extracted with 1 mL chloroform methanol solution. After sample preparation, GC-MS (7890A/5975C, Agilent Technologies, Santa Clara, CA, USA) was used to detect the free acids in the extract. Finally, the chromatographic peak area and retention time were extracted using the MSD Chemstation software (G1701DA, Agilent Technologies, Santa Clara, CA, USA), and the fatty acid content was calculated using a standard curve. SCD activity was estimated by the ratios of C16:1N7/C16 and C18:1N9/C18. The analysis of hepatic free acids by targeted metabolomics was performed by Apply Protein Technology (Shanghai, China).

### 4.5. SCD1^−/−^ Mice for Verification Experiment

*SCD1^−/−^* mice were used to evaluate the role of SCD in improving liver steatosis through 6-G treatment. As described in our previous study [47], *SCD1^−/−^* mice and the wild type (WT) littermates (*SCD1^+/+^*) with a C57BL/6J background were generated by the Nanjing Biomedical Research Institute of Nanjing University (stock No: T002801; name: B6/NjuSCD1em1Cd/Nju). A total of 18 WT mice (8-10 weeks old, 9 female and 9 male) and 17 *SCD1^−/−^* mice (9-12 weeks old, 8 female and 9 male) were used in this experiment (information concerning the *SCD1^−/−^* mice can be found in our previous study [43]). These mice were divided into six groups as follows: (1) WT group (n = 6, *SCD^+/+^* mice, fed a normal diet); (2) FRU group (n = 6, *SCD1^+/+^* mice, fed a high-fructose diet); (3) FRU + 6-G group (n = 6, *SCD1^+/+^* mice, fed a high-fructose diet and administrated 6-G, 2 mg/kg, i.g); (4) KO group (n = 5, *SCD1^−/−^* mice, fed a high-fructose diet); (5) KO + OA group (n = 6, *SCD1^−/−^* mice, fed a high fructose diet supplemented with OA, where OA constitutes 20% of the diet weight); (6) KO + OA + 6-G group (n = 6, *SCD1^−/−^* mice, fed the same diet as the KO + OA group and administrated 2 mg/kg 6-G, i.g). High-fructose diets were purchased from Sigma Aldrich (60% fructose, Cat^#^ 364525, Shanghai, China), 6-G was dissolved in 5% gum arabic solution. According to the grouping described above, mice were gavaged once a day with 6-G or 5% gum arabic solution, respectively. The experiment lasted for 14 days, after which, serum and liver tissue samples were collected for further research.

### 4.6. Quantitative Real-Time Polymerase Chain Reaction (qRT-PCR) Analysis

Quantitative real-time polymerase chain reaction (qRT-PCR) analysis was conducted to evaluate the key genes in liver and HepG2 cells following 6-G treatment. Briefly, total RNA was extracted from livers or HepG2 using TRIzol^®^ reagent (Invitrogen, Carlsbad, CA, USA), and reverse-transcribed using the TaqMan Reverse Transcription Reagents (Applied Biosystems, Foster City, CA, USA). Quantitative real-time PCR was performed with SYBR Premix Plus (Tiangen Biotech Co., Ltd., Beijing, China) according to the manufacturer’s instructions. The gene-specific primers are listed in Supplemental Table S3. Relative mRNA expression was determined by a comparative method (2^−ΔΔCt^), with β-ACTIN serving as a reference gene.

### 4.7. Western Blot Assay

Total proteins were extracted from rat liver and HepG2 cells, separated by 10% SDS−PAGE, and transferred onto polyvinylidene fluoride (PVDF) membranes. The membranes were blocked with 5% skimmed milk for 2 h, and then incubated overnight at 4 °C with primary antibodies against SCD (1:1000, CAT# 28678-1-AP, purchased from Proteintech Group, Inc., Wuhan, China), β-ACTIN (1:3000, CAT# 4967, Cell Signaling Technology, Beverly, MA, USA), AMPK (1:3000, CAT# 2532, Cell Signaling Technology, Beverly, MA, USA), p-AMPK (Thr172) (1:2500, CAT# 2535, Cell Signaling Technology, Beverly, MA, USA). CPT1α (1:3000, CAT# ab128568, Abcam, Cambridge, UK). Next day, after washing with TBST (containing 0.1% Tween-20), the membranes were incubated with an HRP-conjugated secondary antibody (Beijing Biosynthesis Biotechnology, Beijing, China) for 1 h at room temperature. After that, the membranes were washed three times with TBST. Finally, the protein expression was detected with chemiluminescence (Bio-Rad, Hercules, CA, USA).

### 4.8. Statistical Analysis

Data are expressed as mean ± SEM (animal samples in vivo, at least five samples each group) and mean ± SD (cell samples in vitro, from at least three independent experiments). Comparisons were performed using one-way ANOVA for multiple groups or the Student’s *t*-test for two groups (GraphPad Prism 8.0, San Diego, CA, USA). *p* < 0.05 was considered statistically significant.

## 5. Conclusions

In conclusion, our research suggests that 6-G mitigates hepatic DNL by targeting SCD to ameliorate fructose-induced hepatic steatosis. These findings offer a novel perspective on the therapeutic potential role of 6-G (or ginger) in treating MAFLD. Furthermore, our study may provide valuable dietary guidelines for MAFLD patients in clinical practice.

## Data Availability

The data presented in this study are available on request from the corresponding author.

## Supplementary Materials

The supporting information can be downloaded at: https://www.mdpi.com/article/10.3390/ijms252011289/s1.

## Abbreviations

6-G: 6-gingerol; AMPK: adenosine 5′-monophosphate (AMP)-activated protein kinase; CPT1α: carnitine palmitoyl transferase 1α; CETSA: cellular thermal shift assay; DNL: de novo lipogenesis; HDL-c: low density lipoprotein cholesterol; HF: high fructose; HFD: high-fructose diets; LDL-c: low-density lipoprotein cholesterol; MASLD: metabolic-associated fatty liver disease; NAFLD: non-alcoholic fatty liver disease; NASH: non-alcoholic steatohepatitis; OA: oleic acid; SCD: stearoyl-CoA desaturase; TC: total cholesterol; TCM: traditional Chinese medicine; TG: triglyceride.
