# Supplementary material for: 6-Gingerol Inhibits De Novo Lipogenesis by Targeting Stearoyl-CoA Desaturase to Alleviate Fructose-Induced Hepatic Steatosis
Source: Int J Mol Sci. 2024 Oct 20;25(20):11289. doi: 10.3390/ijms252011289 (PMC11508832; doi:10.3390/ijms252011289)
Supplement: Supplementary file 1 [file ijms-25-11289-s001.zip › Supplemental Material.pdf]

Supplemental Material

1. Supplemental Tables

Table S1: The identified protein targets by CETSA-Proteomics approach. And it has been shown in additional EXCEL file named Table S1

Table S2: The representative content of free fatty acids. And it has been shown in additional EXCEL file named Table S2

Table S3 sequences of siRNA and primers

Sequences		
Name	Species	Sequences
SCD-siRNA	Human	Forward:GGAGAAACAUCAUCCUUAUTT<br>Reverse: AUAAGGAUGAUGUUUCUCCTT
NC-siRNA	Human	Forward:UUCUCCGAACGUGUCACGUTT<br>Reverse: ACGUGACACGUUCGGAGAATT
SCD primer	Human	Forward: CTGGAAAGTGATCCCGGC<br>Reverse: GCATCGTCTCCAACTTATCTCC
ACTIN primer	Human	Forward: ACCTTCTACAATGAGCTGCG<br>Reverse: CCTGGATAGCAACGTACATGG
SCD primer	Rat	Forward: CAGTTCCTACACGACCACCACTA<br>Reverse: GGACGGATGTCTTCTTCCAGAT
GAPDH primer	Rat	Forward: GAAGGTCGGTGTGAACGGAT<br>Reverse: CCCATTTGATGTTAGCGGGAT
SCD primer	Mice	Forward: CAGTTCCTACACGACCACCACTA<br>Reverse: GGACGGATGTCTTCTTCCAGAT
ACTIN primer	Mice	Forward: ACCTTCTACAATGAGCTGCG<br>Reverse: CCTGGATAGCAACGTACATGG

2. Supplemental Figures

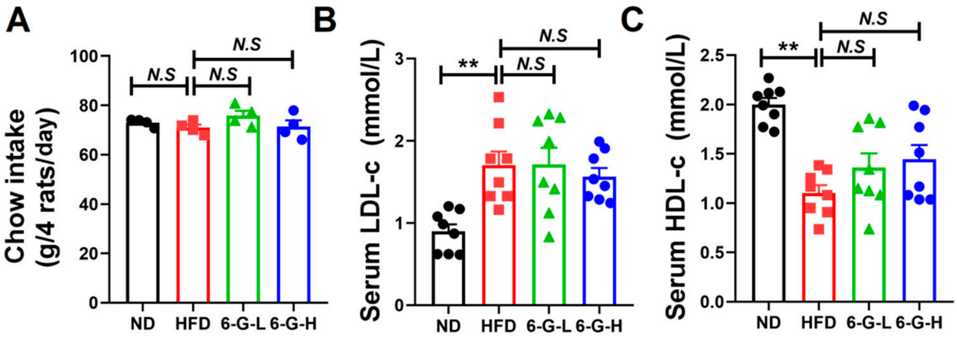

Figure S1. Chow intake, serum LDL-c and HDL-c of HFD-induced rats. A: chow intake, 4 rats were raised in a cage, then measured and counted the intake consumption of each cage per day; B: Serum LDL-c; C: Serum HDL-c. Data are expressed as Mean ± SEM, *n*=8 rats per group. $^{**}p < 0.01$, $^{*}p < 0.05$. N.S: no significant differences.

^{1}H-NMR and ^{13}C-NMR were used for characterized with AI-6G, data and the spectrum were shown as following: HR-MS showed the molecular weight was 332.21 Da, ^{1}H-NMR (600 MHz, $CDCl_3$) δ 6.94 (s, 1H), 6.71 (s, 2H), 4.73 (s, 2H), 4.02 (s, 1H), 3.86 (s, 3H), 2.90 (s, 1H), 2.84 (s, 2H), 2.75 (s, 2H), 2.59 (d, J = 2.8 Hz, 1H), 2.56 (d, J = 2.8 Hz, 1H), 2.48 (s, 2H), 1.47 (s, 1H), 1.37 (s, 2H), 1.28 (s, 5H), 0.89 (s, 3H)；^{13}C-NMR (151 MHz, $CDCl_3$) δ 211.28, 149.69, 145.19, 134.98, 119.97, 114.72, 112.18, 78.73, 75.65, 67.67, 56.91, 55.88, 49.35, 45.23, 36.45, 31.74, 29.20, 25.14, 22.60, 14.03.

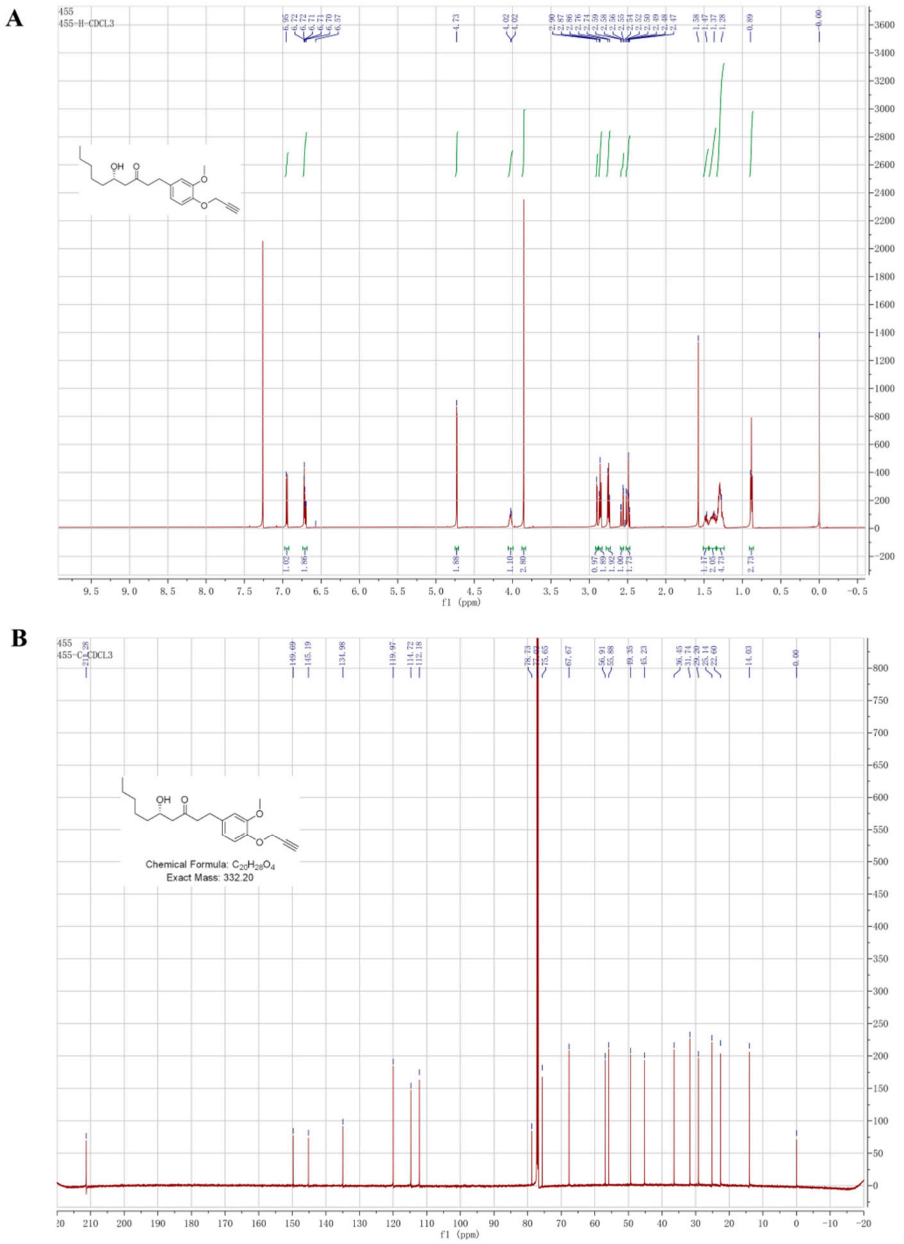

Figure S2. ^{1}H-NMR and ^{13}C-NMR spectra of Al-6G. A: ^{1}H-NMR; B:^{13}C-NMR

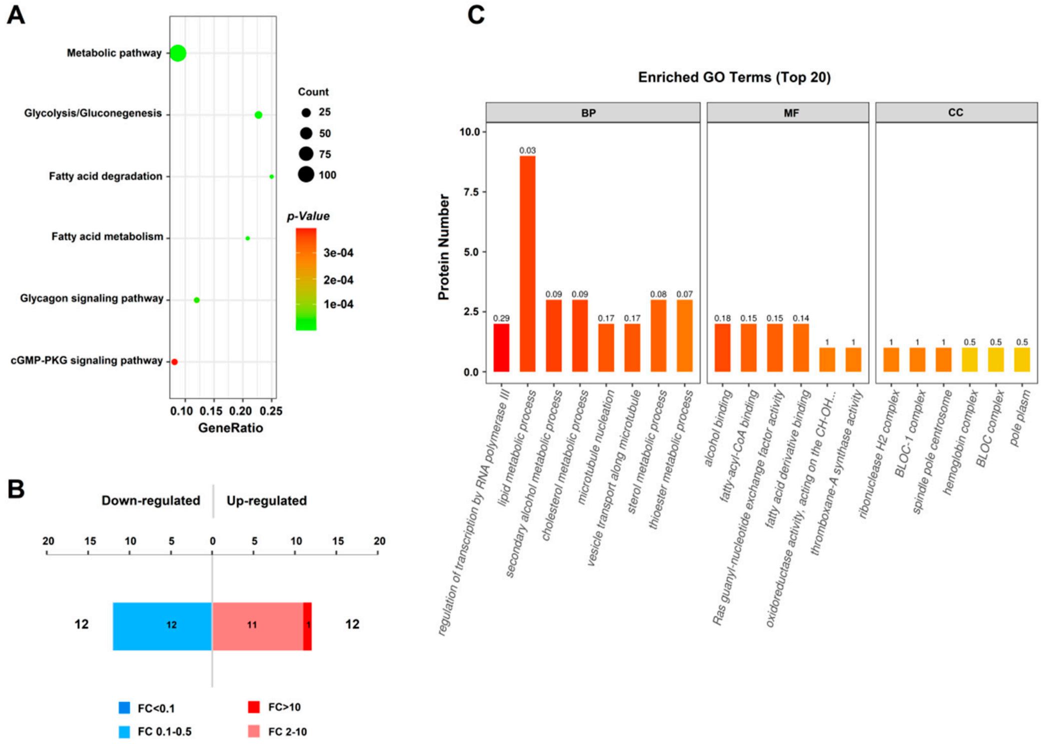

Figure S3. Analysis of the direct binding targets. A: Enrichment pathway analysis of the targets identified by chemical proteomics (KEGG); B: Targets identified by CETSA-proteomics; C: GO analysis of these targets identified by CETSA-proteomics approach.

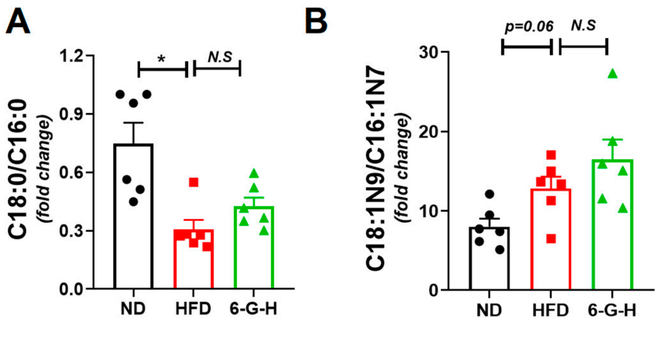

Figure S4. Effect of 6-G treatment on the C18:0/C16:0 and C18:1N9/C16:1N7 ratios. A: C18:0/C16:0 ratio; B: C18:1N9/C16:1N7 ratio. Data are expressed as Mean ± SEM, *n*=6 rats per group. $^{**}p < 0.01$, $^{*}p < 0.05$. N.S: no significant differences.
